# Supplementary material for: Unlocking the Potential of mHealth: Integrating Behaviour Change Techniques in Hypertension App Design
Source: Int J Environ Res Public Health. 2025 Sep 25;22(10):1487. doi: 10.3390/ijerph22101487 (PMC12563110; doi:10.3390/ijerph22101487)
Supplement: Supplementary file 1 [file ijerph-22-01487-s001.zip › Supplementary Table S2- proof.pdf]

Supplementary Table S2

Table S2. MARS dimensions assessment on included apps.

App Name	App type	Engagement Score	Functionality Score	Aesthetic Score	Information Score
Lark	AI	4.4	5	4.67	4.86
CardioX	AI	3	4.75	5.00	3.14
Aktiia	AI	3.8	4.75	4.33	4.00
Cipra AI	AI	3.8	4.75	4.00	4.43
TensionBot	AI	2.8	3.75	3.33	4.14
HelloHeart	AI	4	5	5.00	4.14
Binah.ai	AI	3.8	5	4.67	4.00
Blood Pressure AppSmart BP	Non-AI	3.8	5	4.67	4.14
Blood Pressure Companion	Non-AI	2.6	5	3.00	3.57
BP Wiz Pro	Non-AI	3.8	4.75	4.00	3.86
Braun Healthy Heart	Non-AI	3.2	4.75	4.67	3.71
Cora- Blood Pressure	Non-AI	4.8	5	5.00	4.29
Fast BP Blood Pressure Log & Tracker	Non-AI	3	5	3.33	3.43
HeartStar BP Monitor	Non-AI	4	4.75	4.67	3.86
Blood Pressure Diary	Non-AI	3.2	3.75	4.00	3.71
Cardio Journal- Blood Pressure Diary	Non-AI	4	2.75	4.33	3.71
EHS care	Non-AI	3.2	5	3.00	4.29
