# Supplementary material for: Unlocking the Potential of mHealth: Integrating Behaviour Change Techniques in Hypertension App Design
Source: Int J Environ Res Public Health. 2025 Sep 25;22(10):1487. doi: 10.3390/ijerph22101487 (PMC12563110; doi:10.3390/ijerph22101487)
Supplement: Supplementary file 1 [file ijerph-22-01487-s001.zip › Supplementary Table S1- proof.pdf]

Supplementary Table S1

Table S1. Summary of Characteristics of reviewed apps. N: No; Y: Yes; NA: Not Available.

App Name	Developer	Number of Downloads	Release Date	Last Update	Platform (iOS/Android/Both)	Free	Paid	Primary Function	User Ratings	AI	Wearable
AI-empowerment App											
Lark	Lark	100,000	NA	NA	Both	X	X		3.9	Machine Learning	Y
CardioX AI	CARDIOX INC	1,000	Aug-21	Aug-23	Both	X		Estimate BP	NA	Machine Learning	Y
Aktiia	Aktiia	10,000		13/05/2024	Both		X	Monitor BP	4.4	Machine Learning	Y
Cipra AI	Cipra	100		05/04/2024	Both				NA		
Tensionbot (Telegram) (chatbox)	Telegram	NA	2018	NA	Both			Monitor BP	NA	AI chatbot	N
HelloHeart	HelloHeart	100,000		16/05/2024	Both			Monitor BP	NA	Machine Learning	Y
Binah.ai	Binah.ai	NA			iOS		X	Monitor vital signals	4.8	Machine Learning	N
Non-AI-based App											
Blood Pressure App SmartBP	Evolve Medical Systems LLC	NA		NA	Both	X		Monitor BP	4.5	NA	Y
Blood Pressure Companion	Maxwell Software	NA		01/11/2023	iOS	X		Monitor BP	4.3	NA	N

BP Wiz Pro - Blood Pressure Companion and Drug	LinkLinks LTD	NA	2014	NA	iOS		X	Monitor BP and medication	3.9	NA	N
Braun Healthy Heart	Kaz USA, Inc	100,000	2015	2023	Both	X		Monitor BP	4.2	NA	N
Cora- Blood Pressure	Swiftware	NA	2019	2024	iOS	X		Monitor BP	4.4	NA	Y
Fast BP – Blood Pressure Log & Tracker	Christion Richert	NA		2020	iOS		X	Monitor BP	5	NA	N
HeartStar BP Monitor	Pattern Health	NA		2021	iOS	X		Monitor BP	4	NA	Y
Blood Pressure Diary	FRUCT	1,000,000	2018	2024	Android	X		Monitor BP	4.8	NA	N
Cardio Journal- Blood Pressure Diary	mEL Studio	1,000,000	2017	2024	Android	X		Monitor BP	3.8	NA	Y
EHS care	YouCo	10,000	2016	2023	Android	X		Monitor BP	NA	NA	N
